# Leadership, Management and Command in the time of the Coronavirus

**DOI:** 10.1177/1742715020922445

**Published:** 2020-04-23

**Authors:** Keith Grint

**Affiliations:** Warwick University, UK

**Keywords:** Leadership, Management, Command, Coronavirus, role

## Abstract

The Covid-19 pandemic that swept through the world in late 2019 and through 2020 provides a test not just for all societies and their leadership, but for leadership theory. In a world turned upside down, when many conventions are disposed of, it is clear that things will not return to the status quo ante any time soon, if ever. In the light of these challenges, this short paper suggests we might reconsider the way governments and their leaders act against the frame of societal problems, originally established by Rittell and Webber in 1973. I suggest that all three modes of decision-making (Leadership, Management and Command) are necessary because of the complex and complicated nature of the problem and conclude that while Command is appropriate for certain times and issues, it also poses long-term threats, especially if the context is ignored.

Henrik Ibsen’s *An Enemy of the People* was written in 1882, after his previous play *Ghosts* had received poor reviews for its attack upon the hypocrisy of public morality.^1^ Ibsen wanted to respond by illustrating the way that telling the truth was both necessary, and often unpopular, and that direct democracy had its limitations.

Few people like to hear bad news, especially from their leaders in bad times, when we all seek solace and comfort. But telling people good news is easy, even (or especially) if it is not true; while telling people things they need to hear that they would rather not, is much more difficult, and therefore a more important test of leadership. In *An Enemy of the People*, the bad news is that the new public baths have been poisoned by the local tannery, just as the tourist season is starting (this, of course, is the frame for the 1975 Spielberg movie ‘Jaws’). In the play, the hero, Dr Stockmann fails to persuade his brother, the mayor, to close the baths and is then shouted down at a town meeting for trying to persuade the people that they have an unpopular but necessary duty to perform; they call him ‘the enemy of the people.’ This is the opposite of telling people lies that keep followers happy. And it might be no coincidence that one of the heroes of the UK’s Prime Minister, Boris Johnson, is Larry Vaughn, the Mayor of Amity in Jaws, who wants to keep the beaches open, despite the evidence that a shark is devouring the swimmers one by one (Heritage, 2020).

In our own Coronavirus times, the equivalent problem is represented by both Johnson and Trump beginning the response to the threat by denying its potential significance and ignoring their own advice about social distancing and wearing face masks. Then, when reality threatened to swamp their respective medical systems, switching from Larry Vaughn to Martin Brody, the Chief of Police in Amity, suddenly demanding that all the metaphorical beaches be closed, telling people that they have to quarantine themselves, stop going to work or clubs and bars, and stay at home to protect themselves, their neighbours and their health system. But here is the thing: even democratic leadership is not necessarily about popularity. It is about doing what is right, even if that means sacrificing your own popularity and career. And confidence is not the equivalent of competence. There are times for optimism and times for realism, and sometimes the quest for popularity undermines the importance of being realistic rather than over-optimistic.

We can perhaps understand the role of leadership better if we situate the whole Coronavirus issue in the Tame/Wicked/Critical problems frame (Grint, 2005). Tame Problems can be complicated, but they are solvable through standard operating procedures and are the remit of experts, this is the land of Management. We might put the testing of Covid-19 in this category – we know, theoretically, how to do it, as long as the kits and experts have been made available. Wicked Problems are complex, may not be solvable, but might be ameliorated with a collective response, so the job of Leadership in these circumstances is to mobilize the community, often to address issues they would rather not contemplate. In this category, we could put asking communities to self-isolate and help each other when food and medical supplies are scarce – and no-one quite knows what to do. Critical Problems are crises that need a commander to coerce her or his followers into line to avoid a catastrophe – ordering schools and business to shut for the foreseeable future would be an example of this decision-making.

In effect, if we get the wrong decision mode for the problem that we are facing, then we are likely to make it worse: hence indecision about what to do and seeking collective ideas when the virus is already upon us is to adopt Leadership when Command is required. Likewise, ordering businesses to close without understanding or caring about the wider impact upon the community in the absence of state-provided income, is to confuse the importance of collaborative Leadership with the apparent decisiveness of Command. But you can be decisively wrong unless you have taken advice from experts on what will happen next. And ignoring those medical experts – while listening to the self-interests of those with fortunes at stake in a flailing economy – is just as likely to lead to chaos.

The role of Leadership is to get communities on board to help themselves wherever possible, and make people face things that have to be faced, like social distancing and quarantine, but to ensure that these decisions are done equitably and without displacing blame onto scapegoats when responsibility lies much closer to home.

And the role of Command is to take the tough decisions when groups and individuals persist in anti-social and illegitimate behaviour at a time of crisis. It is self-evident that coercive legislation, in and of itself, is unable to change people’s behaviour. There have always been, and always will be, errant individuals that flout rules designed to protect majorities or minorities, but that is not the role of such legislation. As Martin Luther King suggested in a speech on 7 December 1964 in London, ‘It may be true that the law can’t change the heart, but it can restrain the heartless.’

We all need our political leaders to play the Dr Stockmann, to tell us the truth, even if it is unbearable, and to tell us our responsibilities, even if we would prefer to shirk them. This is the Leadership role. We also need them to support the experts who can test and subdue the virus and comfort those that cannot be protected. Our political leaders also need to engage in the Command mode where we fail to adhere to the advice of experts and refuse to help our communities in their hour of need. But Leadership should not be dictated by popularity, or by serving the minority interests of one part of the community whilst sacrificing those of the majority. Instead, Leadership is doing what is right for the community, even if that means losing an election.

But doing something which is contrary to your own instincts is a tough call. In 1646, as the First English Civil War drew to a close, a song called ‘The World Turned Upside Down’ was written in response both to the war in general, and the banning of Christmas celebrations in particular, and surely it is an appropriate title for the world we currently live in, for many aspects of our daily normality have been turned upside down by the Corona virus. Indeed, it is not just our routines that have disappeared but also what counts as normal in politics, as Prime Minister Johnson and President Trump, both avid adherents of free market philosophies, engage in state bailouts, income support and centralised controls that would not look amiss in the Soviet Union of old or the contemporary regime in China.

And yet, despite the radical about-face in the USA and the UK, as the free market has shown its limits, the virus has also revealed some of the contemporary myths which we live by, to be equally suspect. Both the UK and the USA have taken different strategic responses compared to either most of their European allies or even their global competitors, often on the basis that the Anglo-Saxon cultures are ‘exceptional’ by comparison to others – i.e. superior to them. The empirical data suggest that they are exceptional, but often in a negative sense, though the full results will only be clear when this is all over. Partly this is rooted in what David Collinson (2012) called the ‘Prozac Leadership’ embodied by both leaders and their administrations – the over-emphasis on the positive and the denial of any bad news – and partly it is to do with the failure to understand the nature of the problem facing them, and thus to take an inappropriate and inadequate response. The former is a personality issue, the latter is a more systemic problem because the governments of both Johnson and Trump have decried the value of science and experts for so long that the volte-face required to bring science back to the political stage has proved difficult. Indeed, the U turns, claims and counter-claims about the problem and the availability of medical tests and equipment reminds me of Winston Smith, the main character in Orwell’s *1984*, fretting over how many boots he should report that had been delivered: ‘The Ministry of Plenty’s forecast had estimated 145 million pairs. The actual output was given as 62 million … Winston marked the figure down to 57 million … In any case, 62 million was no nearer the truth than 57 million or 145 million.’

And like the Ministry of Plenty’s forecast, the unpleasant truth is that no-one actually knows what is or might be happening because the testing has not been rigorous, and the sampling is not random. This means that when we hear ‘the expert advice’ trotted out, there ought to be a health warning about taking expert advice as ‘the truth’ when in reality we actually mean, the various models employed suggest or imply that X or Y has a greater or lesser likelihood of happening – but we cannot be certain. And the absence of that certainty runs contrary to all we know about what many people seek in a crisis: a commander with the answer, prepared to ‘do whatever it takes’ or ‘make it happen’ or ‘meet again’ or whatever.

Unfortunately, the problem we are looking at is not a Critical Problem with a solution, just requiring a commander to steadfastly see us through this, but a Wicked Problem that has no clear end but rather an array of possibilities, each of which has more or less deleterious consequences. We could hold the lock-down for six months and destroy the economy to the point where the recovery is decades in the making and people’s mental health is dangerously affected; or we could lift the quarantine now and get the economy going but see a significant increase in illness and death. Whatever we do is only partly determined by how we manage ‘the scientific data’ because our data is not very good (Ionaddis, 2020), and partly determined by political interests, because that is what the system runs on. But many of us do not want to hear these truths – we want someone to save us, and as the history of charisma leadership tells us, the person who exudes confidence and appears to have a cast-iron explanation of the problem and solution to it, will probably take the helm, even as the symbolic reef appears over the horizon.

Winston Smith was not alone in either his confusion about the number of boots/ventilators/Covid-19 tests being produced or the nature of ‘the enemy.’ In my own village near Oxford, England, the first rumours of a ‘virus carrier’ led to a social media frenzy as otherwise friendly neighbours went searching online to detect the infected house, in a symbolic reversal of the tale in Exodus 12:13, where blood on the door is taken as a sign to God that the plague will not take those dwelling inside. The idea that hitherto integrated communities could turn on each other so swiftly in times of crisis should not be a surprise: we only need to look at the Balkans War in the 1980s to see how quickly neighbours would ‘cleanse’ themselves of the ‘enemy’ under specific conditions, and this is a reminder of how thin the veneer of civilisation can sometimes be – and why it is the responsibility of political leaders to reinforce our collective struggle against the virus, not against the alleged enemy next door.

This tendency to perceive the ‘enemy within’ as the threat, rather than the actual virus, is compounded by the way leaders adopt particular forms of language. Invoking metaphors of war and calling the population to arms might be essential under threat of an actual enemy invasion, but this is an existential crisis, like the environmental threat but unlike the World Wars. As such, the language should reflect our common human values not distinct nationalist values, because the virus does not have a nationality, is uninterested in political boundaries and unconcerned by border walls: a global pandemic requires a global, not a nationalistic response.

The pandemic has also exposed the good and the bad side of society. Dickens’ *Tale of Two Cities* starts with the following line: ‘It was the best of times, it was the worst of times, it was the age of wisdom, it was the age of foolishness, it was the epoch of belief, it was the epoch of incredulity, it was the season of light, it was the season of darkness, it was the spring of hope, it was the winter of despair.’ And times such as this do indeed bring out the best and worst in us – and always have done. Samuel Pepys allegedly railed against the stupidity of gangs of young London men – ‘a gaggle of striplings’ – during the English plague in 1664 (though Pepys’s quote actually comes from 2020 and was written by someone pretending to write as if they were Pepys; actual fake news). Similarly, the UK has evoked images of the village of Eyam in Derbyshire which, in 1665, quarantined itself to keep the plague within the village, rather than spread it into the surrounding countryside; and yet there are precious few other examples of such collective sacrifice despite it becoming a nationalist symbol of resilience. Exactly the same patterns are visible today, as ambulances in the UK are sometimes attacked by the ignorant while elsewhere legions of volunteers put themselves at risk to help those less fortunate. Indeed, the ‘Keep Calm and Carry On’ slogan that is alleged to have epitomised the spirit of Londoners during the Blitz and the British now, is often touted as a rallying call for those currently under siege. But the slogan did not actually appear until after the Blitz, and while the Blitz did stimulate extraordinary bravery from all kinds of people, it was also the era when ‘spivs’ and charlatans took advantage of the chaos to loot bombed-out houses and even carry out vendettas against their personal rivals (Calder, 1992; Overy, 2020).

The role of leadership, then, is not to pretend that the unique values of the country will save it, but to support those that need help, and suppress those that remain irresponsible, for whatever reasons, while supporting the research into vaccinations and the health systems that need experts to survive. That judicious combination of manager, commander and leader is never simple, but it is necessary. In Trump’s case, the commander is often decisive, but the direction changes dependent upon the polls. In Johnson, a man hitherto famous for the attributions of a clown rather than a commander, we British have a leader who has had great difficulty becoming the commander.

But the most dangerous contemporary commander seems to be the Indian Prime Minister, Modi, whose decision to quarantine the country with just a few hours’ notice, and with little apparent planning for the consequences of the decision on the poorest members of Indian society, has seen a mass exodus from the cities that can only make the problem worse. And this example reminds us to locate decisions in the appropriate context: in countries without a relatively sophisticated health system or the necessary social infrastructure, simply commanding people to stay home without providing any means for them to survive more than a few days, or instructing them to self-isolate, makes little sense. Indeed, as Capt. Brett Crozier, until recently the commanding officer of the aircraft carrier USS *Theodore Roosevelt* (CVN 71), has found to his cost, such ships do not provide appropriate conditions for containing an outbreak of Covid-19.^2^ As the man who dismissed Crozier so decisively, US Navy Secretary, Thomas Modly, has discovered, when being decisive, as a Commander should be, it is important not to be decisively wrong.

## Conclusion

The arrival of Covid-19 has turned the world upside down for many of us, and when it stabilises, it will not be exactly as it was, though what it might look like is not determined by events unfolding before us. The pandemic has exposed the limits of market-based economic and health systems but also threatens to engulf even the most egalitarian societies. And in times of crisis, it appears that many of us seek out charismatic leaders or authoritarians who can, allegedly, defeat the virus just by remaining positive or simply by denying the evidence, confusing as that is. If leadership is partly about making people face up to unfortunate truths – and it surely is – then we need leadership that embodies this, at the same time as we manage the research and resources to keep the systems going. Of course, we also need commanders when and where necessary, but all of these decision-modes need to be contextualised and deployed carefully. And we need to be especially wary of commanders who seek to persuade us that ‘the situation’ – which they cannot reveal for fear of frightening us further – is so dire that they need to continue the state of emergency, ‘for our own safety.’ Otherwise, as Winston Smith persuades himself at the end of *1984*, he was previously an enemy of the people but now he does, after all, ‘love Big Brother.’

## Notes

1 A version of this was first published as a blog on the International Leadership Association’s website on 30 March 2020. www.ila-net.org/Reflections/index.html.

2 www.bbc.co.uk/news/world-us-canada-52145230 (accessed 8 April 2020).
